# Factors associated with intimacy and sexuality among young women with multiple sclerosis

**DOI:** 10.1186/s12978-020-00960-5

**Published:** 2020-07-10

**Authors:** Khadijeh Mohammadi, Parvin Rahnama, Zahra Rafei, Seyedeh Mitra Ebrahimi-Aveh, Ali Montazeri

**Affiliations:** 1 Department of Midwifery, Faculty of Nursing and Midwifery, Shahed University, Tehran, Iran; 2 Independent Research Fellow, Tehran, Iran; 3 Population Health Research Group, Health Metrics Research Centre, Iranian Institute for Health Sciences Research, ACECR, Tehran, Iran; 4 Faculty of Humanity Sciences, University of Science &Culture, ACECR, Tehran, Iran

**Keywords:** Multiple sclerosis, Multiple sclerosis intimacy and sexuality Questionnaire-19 (MSISQ-19), Sexual dysfunction

## Abstract

**Background:**

Patients with multiple sclerosis (MS) especially those with younger age experience an alteration in physiological and emotional lifestyle that can affect intimacy and sexuality**.** The aim of this study was to determine demographic and disease related determinants of intimacy and sexuality in young women with MS.

**Methods:**

This was a cross sectional study carried out in Tehran, Iran. A convenience sample of young women aged ≤35 years old with MS attending to outpatient clinics in a teaching hospital affiliated to Tehran University of Medical Sciences was entered into the study. The Multiple Sclerosis Intimacy and Sexuality Questionnaire-19 (MSISQ-19) was used to evaluate how the disease influences sexual function and satisfaction in these patients. Multivariable analysis using hierarchical method was performed to identify variables that are associated with intimacy and sexuality.

**Results:**

In total 117 young women with confirmed diagnosis of MS were included in the study. Participants mean was 25.7 (SD = 8.07) years. A multivariable hierarchical regression analysis was performed using demographic variables entered in step one, relevant neurological variables in step two, and psychological variables in step three. Furthermore, we loaded antidepressant use in the last step. Overall, the seven variables accounted for 39% of total variance observed for the MSISQ score (*P* < 0.001). At step one the demographic variables accounted for 13% of the variance in the MSISQ score (*P* < 0.001). At step two the inclusion of relevant neurological variables increased the R^2^ significantly and explained 27% of variance for the MSISQ (P < 0.001). However in the third step the inclusion of psychological factors increased R^2^ significantly (adjusted R^2^ increased to 0.39, P < 0.001).

**Conclusions:**

The findings indicated that psychological, disease-related and demographic factors (education and employment status) contributed to intimacy and sexuality in young women with multiple sclerosis. Appropriate interventions, especially psychological interventions, appear to be essential immediately following a definite MS diagnosis in young women.

## Plain English summary

A cross sectional study was conducted to ass intimacy and sexuality among young women with multiple sclerosis. Indeed a sample of young patients completed the Multiple Sclerosis Intimacy and Sexuality Questionnaire-19 (MSISQ-19). Then statistical analysis carried out to identify variables that are associated with intimacy and sexuality. The results indicated that education, employment status, disease course and depression significantly were associated with intimacy and sexual satisfaction. The findings suggested that implementing appropriate interventions especially psychological interventions immediately following a definite MS diagnosis might help to improve sexual function in young women suffering from multiple sclerosis.

## Background

Patients with multiple sclerosis (MS) usually experience an alteration in physiological and emotional health that can affect women’s sexual life. MS can influence sexual satisfaction and sexual activity that are often underreported and under-diagnosed [1].. Patients with MS might experience sexual dysfunction, sexual dissatisfaction, and problems in relationships with their partners. These even could be more problematic among younger women who suffer from the disease since apparently they usually have more desire for sexual activity. Overall, the prevalence of sexual dysfunction among patients with multiple sclerosis (MS) is high [2]. The prevalence varies from 40 to 80% in women. For instance a recent publication with a large sample of women (*n* = 1663) from 54 countries found that the majority of patients (55.6%) reported one or more problems with sexual function, and lack of sexual interest (41.8%) [3]. Indeed sexual dysfunction may influence women’s reproductive health and quality of life while they receive no help from health professionals [4, 5].

There are several reasons that health care providers are unable to address sexual function in this community including presence of family or friends, not being asked, and inadequate time during the clinical visits [6]. However, recently we are witnessing growing concern on sexual dysfunction in women with multiple sclerosis especially among women with younger age. As such measuring sexual dysfunction in these patients is increasingly becoming a topic. For instance, recently a MS-specific scale (the MSISQ) was developed to assess the perceived influence of MS symptoms on the overall quality of an individual’s intimate relationship [7] or it has been realized that similar to all other human beings MS patients’ desires including sexual needs should be met.

Earlier studies attributed sexual problems in MS to several disease-related problems. It was shown that the neurological impairment measured by the Expanded Disability Status Scale (EDSS) in MS patients contributes to the development of sexual dysfunction (SD) [8, 9]. In fact it was indicated that neurological impairments from the sacral segments including weakness of the pelvic floor and bladder and bowel dysfunction affect sexual function [10]. Others stressed the role of psychological variables such as depression. It was reported that there was significant correlation between the neuropsychological consequence of MS such as depression and sexual dysfunction [9]. A recent review of the literature concluded that there is an urgent need for the development and adequate evaluation of psychological interventions for sexual dysfunctions in MS. The authors suggest that sexual dysfunction and its impact on psychological health should be more focused in practice [11].

This study was conducted to evaluate demographic and disease related determinants of intimacy and sexuality in young women with MS. Associations between sexual function in women with multiple sclerosis and demographic, disease and lifestyle characteristics are well documented. It has been suggested that sexual dysfunction and lack of satisfaction with sexual function among people with multiple sclerosis are associated with depression, fatigue, as well as modifiable lifestyle factors such as diet and physical activity [3]. In fact it is argued that since patients with multiple sclerosis experience continued fatigue, they feel depressed and such depression in addition to other physical limitations contribute proper sexual function and satisfaction. However, it was hoped that this study might contribute to informing future interventions. Health care officials and health care personnel need to know about the risk factors for sexuality and couples’ relationships. In Iran where about 31% of women are at reproductive age, the prevalence of MS is high [12]. It is believed that of these 55% [13] to 81.9% [14] might suffer from sexual dysfunction or even at higher rate (87.1%) due to primary sexual problems [15]. Unfortunately at present no help is offered to women with MS who experience sexual dysfunction or sexual dissatisfaction in Iran.

## Methods

### Design and participants

As part of an investigation on sexual dysfunction in patients with multiple sclerosis, a cross sectional study was performed to assess demographic, disease related and psychological determinants of intimacy and sexuality in women suffering from multiple sclerosis. Indeed a convenience sample of young women with confirmed diagnosis of MS attending to an outpatient clinic at a teaching hospital affiliated to Tehran University of Medical Sciences was entered into the study. The hospital is the largest teaching hospital in Tehran and usually patients from different socio-economic backgrounds attend to its outpatient clinics. They were asked to complete the study questionnaires on one occasion. Informed consent was obtained from participants before administering the questionnaire. The inclusion criteria were: definite diagnosis of MS according to the McDonald Revised criteria [16]; having the Expanded Disability Status Scale (EDSS) score < 8 [17], age ≤ 35, being married, and have been sexually active in the last 6 months. Exclusion criteria were: pre-existing major chronic illnesses, or not having sexual experiences at all.

### Sample size estimation

The following formula was used to estimate the minimum required sample size [18]:

n = Z^2^P(1-P)/d^2^.

where Z for 95% confidence interval is 1.96, *P* = 0.55 (based on the smallest prevalence of sexual dysfunction in Iranian women with multiple sclerosis 55% [13]), and d = 0.1 (precision = 10%). As such we estimated at least 95 young patients would be adequate for the study to have a power of 80% at 5% significant level. However in practice 117 young women with multiple sclerosis participated in the study.

### Measures

The following questionnaires were used to collect data:

1. Demographic characteristics including respondents’ age, education, employment status, disease duration, and relationship status.
2. Clinical features and disease-related symptoms including information on disease duration, and the disease course according to the McDonald revised criteria that are relapsing remitting MS (RRMS), primary progressive MS (PPMS) and secondary progressive MS (SPMS) [16].
3. The severity of neurological impairment was measured using the Expanded Disability Status Scale (EDSS) [17] as a gold standard. The score on the EDSS ranges from 1 to 9.5 where scores from 1.0 to 4.5 specify that patients are fully capable of walking while higher scores indicate that patients are severely impaired.
4. Depression was measured using the Beck Depression Inventory-II (BDI-II) [19]. The BDI-II has been shown to be a psychometrically sound instrument for use among Iranian population [20]. A cut-off point of 19 and higher was used to indicate moderate to severe depression [21].
5. The Multiple Sclerosis Intimacy and Sexuality Questionnaire-19 (MSISQ-19) that includes 19 items evaluating how MS symptoms influences patients’ sexual function and satisfaction over the previous 6 months. It assesses three dimensions of sexual dysfunction: primary, secondary, and tertiary. Each item is rated on a five-point Likert scale ranging from 1 to 5 (1: never; 2: almost never; 3: occasionally; 4: almost always; and 5: always) [7]. Psychometric properties of the Iranian version of MSISQ-19 are well documented [22].

### Statistical analysis

Data were analyzed using the Statistical Package for Social Sciences for Windows (SPSS, v.16.). Descriptive statistics including frequency, percentage, mean and standard deviation were used to describe demographic and clinical information (age, education, employment status, disease duration, disease course, disability score, depression score, and the MSISQ-19). Bivariate analysis of variables was conducted using Pearson’s correlation coefficient and multivariable analyses (hierarchical linear regression analysis) were used to identify variables associated with sexual intimacy and sexual satisfaction as measured by the MSISQ-19 (the independent variable).

## Results

In total 117 young women (aged ≤35 years) diagnosed with MS were included in the study. The mean age of participants was 35.77 years (SD = 8.07). Forty-eight patients (41.1%) completed their formal education at higher level and the mean duration of MS diagnosis was 1.84 (SD = 0.79) years. Most patients had been diagnosed with relapsing remitting MS (RRMS, *n* = 69, 59.0%), and 48 patients (41%) had either primary progressive MS (PPMS) or secondary progressive MS (SPMS). The mean disability score (EDSS) was 2.99 ± 2.17, and it was 17.8 ± 11.1 for depression severity score (BDI). The detailed results are shown in Table 1.

**Table 1:** The characteristics of study sample (*n* = 117)

	No.	%
**Education level**		
Primary/Secondary	69	58.9
Higher	48	41.1
**Employment status**		
House wife	80	68.4
Employed	37	31.6
**Disease duration (years)**		
0–8	60	51.3
≥ 9	57	48.7
Mean (SD)	1.84 (0.79)	–
**Disease course**		
RRMS	69	59.0
PPMS and SPMS	48	41.0
**Treatment for depression**		
Yes	22	18.8
No	95	81.2
**Disability score (EDSS)**		
0–4.5	71	60.7
5–8	46	39.3
Mean (SD)	2.99 (2.17)	
**Beck Depression score (BDI-II)**		
< 19	91	77.8
≥19	26	22.2
Mean (SD)	17.8 (11.1)	
**MSISQ-19 total score**		
Mean (SD)	46.1 (17.5)	

The correlation matrix between demographic and disease-related variables with the MSISQ is shown in Table 2. All variables except treatment for depression showed significant correlation with the MSISQ-19 total score. Specifically, higher correlation was observed for the MSISQ-19 scores with unemployment, lower educational level, being more disable, moderate or severe depression and progressive stage of MS.

**Table 2:** Correlation between demographic, disease-related variables and the MSISQ-19 total score

	MSISQ-19 total score	Age	Education	Employment status	Disease duration (years)	EDSS score	Disease course	BDI-II	Treatment for depression
**MSISQ-19 total score**	1								
**Education**	−0.292^**^	−0.236^**^	1						
**Employment status**	− 0.336^**^	− 0.116	0.512^**^	1					
**Disease duration (years)**	0.291^**^	0.337^**^	−0.097	− 0.092	1				
**EDSS score**	0.373^**^	0.071	−0.078	− 0.161^*^	− 0.290^**^	1			
**Disease course**	0.319^**^	0.194^**^	−0.031	− 0.092	− 0.226^**^	0.496^**^	1		
**BDI-II**	0.466^**^	0.038	−0.133^*^	−0.241^**^	0.126	0.293^**^	0.043	1	
**Treatment for depression**	−0.109	−0.048	0.009	0.097	0.016	0.099	0.088	−0.057	1

* *P* < 0.05

** *P* < 0.01

A multivariable hierarchical regression analysis was conducted with demographic variables entered at step one, relevant neurological variable at step two, and psychological variables at step three. In addition we load antidepressant use into final step. Overall, seven variables accounted for 39% of total variance in the MSISQ (*P* < 0.001). At step one demographic variable found to account for 13% of the variance in the MSISQ (P < 0.001). At step two, inclusion of relevant neurological variables increased the R^2^ significantly and explained 27% of variance in the MSISQ (P < 0.001). However in the third step inclusion of psychological factor increased the adjusted R^2^ significantly (adjusted R^2^ = 39%, P < 0.001). The results are shown in Table 3.

**Table 3:** The results obtained from hierarchical linear regression analysis to assess association between the MSISQ and independent variables (*n* = 226)

	Factor	Regression coefficient ***(β)***	R^**2**^	Adjusted R^**2**^	P
*Step 1*			0.14	0.13	
	**Age**	0.097			0.13
	**Education**	−0.139			0.06
	**Employment status**	−0.254			0.001
*Step 2*			0.28	0.27	
	**Age**	0.000			0.99
	**Education**	−0.156			0.02
	**Employment status**	−0.194			0.004
	**Disability score (EDSS)**	0.204			0.003
	**Disease course**	0.158			0.01
	**Disease duration (years)**	0.163			0.01
*Step 3*			0.40	0.39	
	**Age**	0.002			0.97
	**Education**	−0.153			0.01
	**Employment status**	−0.124			0.05
	**Disability score (EDSS)**	0.088			0.17
	**Disease course**	0.211			0.001
	**Disease duration (years)**	0.147			0.97
	**Beck Depression score (BDI-II)**	0.362			0.01

## Discussion

The findings from this study indicated that demographic variables account for 13% of the variance in MSISQ. When disease related factors included in the model, disability level was significantly associated with sexual dysfunction followed by employment status, disease course, and education level and disease duration. In fact disease related factors improved the model in explaining sexual dysfunction as compared to previous model.

In our study sexual dysfunction was associated with depression, health and demographic related factors. Age and disability level were diminished in the presence of depression when performing multivariable regression analysis. The strongest association was observed between sexual dysfunction and depression. A study in MS patients revealed that there is no evidence for an association between age and sexual dysfunction [23]. Furthermore other studies indicated that there is no association between EDSS and sexual function, and sexual dysfunction might be occur even when MS patients do not experience severe disability [24, 25].

We found that there was a relationship between duration of the disease and sexual dysfunction. This result is consistent with a number of longitudinal studies showing that sexual dysfunction; frequency and extent of symptoms could increase sexual dysfunction over time [26, 27]. It seems that nature of the disease and fear about the future might leaded to observe such condition. In addition increasing dependence upon to their spouses in women with MS may be affected sexual dysfunction over the time.

The findings showed that women with progressive stage experienced higher rate of sexual dysfunction compared with women at RRMS stage. These findings are consistent with previous studies that also showed that there are correlations between course of study and sexual dysfunction [3, 28].

The etiology of depression in MS patient is multifactorial and the incidence of depression in MS patient increased dramatically recently especially in Iranian women [12, 29]. However, the relationship between sexual dysfunction and depression in patients with multiple sclerosis remains controversial. While similar to our findings some investigators believe there is a direct relationship between sexual dysfunction and depression in multiple sclerosis [9, 25], a recent study reported that sexual dysfunction was linked to disease subtype and disability but depression was not directly associated with sexual functioning [30].

### Limitations

Present study had some limitations including cross sectional nature of the study and sampling from outpatient clinics making difficult to interpret a causal association between intimacy, sexuality and independent variables. Perhaps a better study design is needed to understand this mechanism further. Furthermore small sample size and the use of a hospital-based sampling limit the findings to be generalized for this population.

## Conclusions

The findings indicated that psychological, disease-related and demographic factors (education and employment status) were contributing factors to sexual intimacy and sexuality in young women with multiple sclerosis and the pattern is very similar to adult women experiencing the disease. Since young women with multiple sclerosis experience sexual dysfunction at low disability level therefore it is essential that appropriate interventions especially psychological interventions being implemented immediately following definite diagnosis.

## Data Availability

The data are available from Dr. KM on a reasonable request.

## Abbreviations

MS: Multiple sclerosis
EDSS: Expanded Disability Status Scale
BDI-II: Beck Depression Inventory-II
MSISQ-19: Multiple Sclerosis Intimacy and Sexuality Questionnaire-19
RRMS: Relapsing remitting MS
PPMS: Primary progressive MS and
SPMS: Secondary progressive MS
